# Prevalence and Factors Associated with Musculoskeletal Disorders among Thai Burley Tobacco Farmers

**DOI:** 10.3390/ijerph19116779

**Published:** 2022-06-01

**Authors:** Amarin Kongtawelert, Bryan Buchholz, Dusit Sujitrarath, Wisanti Laohaudomchok, Pornpimol Kongtip, Susan Woskie

**Affiliations:** 1 Department of Occupational Health and Safety, Faculty of Public Health, Mahidol University, 420/1 Rajvidhi Road, Bangkok 10400, Thailand; wisanti.lhk@mahidol.ac.th (W.L.); pornpimol.kon@mahidol.ac.th (P.K.); 2 Department of Biomedical Engineering, University of Massachusetts Lowell, One University Ave, Lowell, MA 01854, USA; bryan_buchholz@uml.edu; 3 Department of Epidemiology, Faculty of Public Health, Mahidol University, 420/1 Rajvidhi Road, Bangkok 10400, Thailand; phdsj@hotmail.com; 4 Department of Public Health, University of Massachusetts Lowell, One University Ave, Lowell, MA 01854-2867, USA; susan_woskie@uml.edu

**Keywords:** Burley tobacco farmers, ergonomics, musculoskeletal disorders, Thailand

## Abstract

This cross-sectional analysis study aimed to identify the prevalence and factors associated with musculoskeletal disorders (MSDs) among Thai Burley tobacco farmers. Subjects included 603 burley tobacco farmers from Sukhothai province. Farmers were interviewed twice, (during planting and harvesting seasons), with a questionnaire consisting of demographic and health characteristics, musculoskeletal symptoms, and ergonomic exposure questions. The subjects average age was 49.5 years, more were female (58.5%), most had only a primary education (74.3%), 38% were overweight or obese. Farmers had a significantly higher prevalence of MSDs in the lower back (37.1%), knee (28.7%), shoulder (22.9%), wrist (19.9%), and hip (8.3%) during the harvesting season than in the planting season (*p* < 0.05). Models found that factors influencing MSDs prevalence during planting included long work hours in seedling, tasks such as topping tobacco plants, and using machine tools, after controlling for age, gender, and body mass index (BMI). While in the harvesting season, models found tasks conducted as a group had lower MSDs prevalence than individual work when carrying fresh tobacco to the barn, piercing/threading and curing the leaves, baling the bundles, and transporting the finished goods. We recommended working in groups to reduce workload and MSDs, especially during harvesting, in burley tobacco farming.

## 1. Introduction

The International Labour Organization (ILO) estimates that 1.3 billion people are employed in the agricultural sector worldwide, which accounts for a large portion of the total labor force, particularly in developing countries. In terms of fatalities, injuries, and work-related ill-health, the agriculture sector is one of the three most hazardous types of work, along with construction and mining [1]. The differences in environmental conditions, such as climate, soil conditions, topography, and type of crop production may place agricultural workers at increased risk for injuries and musculoskeletal disorders (MSDs) [2]. MSDs are defined as injuries or pain that affect the musculoskeletal system, including the nerves, tendons, and muscles, and its supporting structures, such as intervertebral discs [3]. Many studies have shown that MSDs constitute a significant occupational problem among working populations [4,5,6].

At the end of 2021, the total population of Thailand was 66.17 million people. Of this number, 37.7 million were in the workforce, and approximately 10.8 million engaged in the agricultural sector [7]. A few studies of MSDs among agricultural workers have been conducted in Thailand, including maize farmers [8], rice farmers [9], sugarcane farmers [10], and rubber tappers [11]. It is estimated that Thai agricultural workers’ occupational injury and illness cost was 47 million USD in 2017 [12].

In Thailand, tobacco is considered a significant cash crop for many farmers. More than 40,000 tobacco family farmers plant around 82,000 acres and generating more than 46 million USD per year in the economy by supplying tobacco leaves to the Thailand Tobacco Monopoly (TTM) [13,14]. Any farmers who cultivate tobacco plants must possess a license issued by the Excise Department, Ministry of Finance in Thailand. There are three categories of tobacco cultivation: (1) cultivation under contract with the TTM, (2) self-cultivation for sale to local shredded tobacco factories, and (3) cultivation under contract to private companies for export to other countries.

Three species of tobacco are cultivated in Thailand, namely the Virginia strain, mainly in the north; the Burley strain in the lower north and the northeast; and the Turkish or oriental strain in the northeast and upper central plain. The most significant production volume is that of the Burley strain. Sukhothai province, located in the lower northern part of Thailand, is one of the most well-known areas for the cultivation of Burley tobacco. In 2015, Sukhothai reported around 18 to 20 million kilograms on 20,000 acres of cultivation.

**Figure 1:**
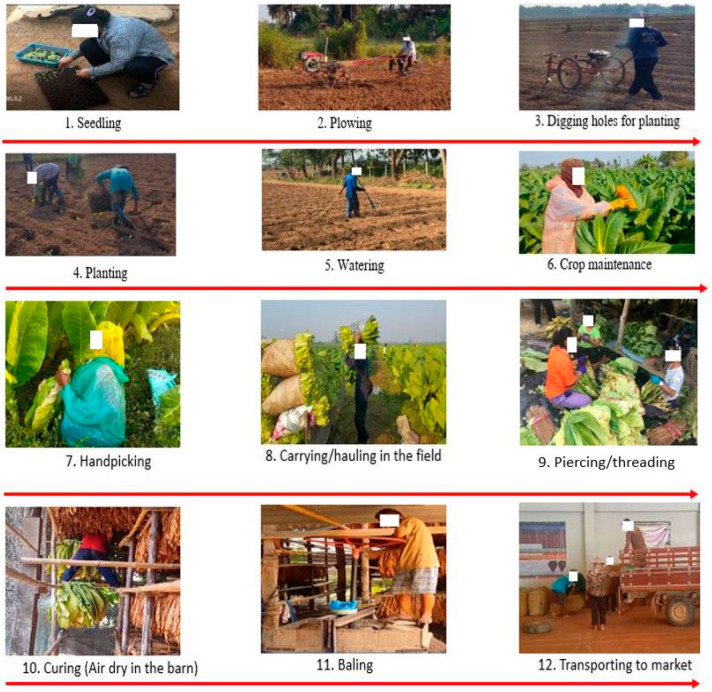
Burley tobacco production: steps 1–6 for the planting season and steps 7–12 for the harvesting period.

Burley tobacco production in Thailand is different from that found in Western countries due to its labor-intensive nature. There is minimal mechanized equipment used throughout the process. Figure 1 shows the steps in tobacco production, starting with the growing period, which usually occurs from November to the end of January.

Growing consists of several tasks. It begins with growing the young tobacco plants or seedlings in a greenhouse (step 1) and then manually transplanting them into the field. Hand tractors and hole digger machines were used for plowing as the soil preparation process (step 2) and making holes to transplant the young tobacco into them (step 3).

While the plants grow in the field for several months, crop maintenance is required. This crop maintenance involves watering the plants, application of fertilizer and pesticides, removal of axillary buds and the blooms from the top of the tobacco plant (topping process), and removal of weeds from the field (steps 5 and 6). The postures of the farmers in planting (pegging or setting) the young tobacco plant to the field involve repetitive stooping and bending at the waist (step 4).

The first crop is ready for harvesting after 60 days, usually from February until May. During this harvesting period, the farmers must start to pick the leaves by hand, beginning from the bottom of the plant and continuing to the top by the end of the harvesting period (step 7). The postures of the farmers in this process include stooping and bending the back to the right level for picking the leaves, twisting the wrists for picking the leaves, and carrying leaves with one arm to load them into wagons (step 8).

Once harvested, the fresh leaves are transported to a barn, where the drying (curing) process takes place. The tasks for the curing (air drying) process include piercing/threading the tobacco leaves onto wooden sticks (60–80 cm or 2–2.5 feet in length) (step 9). For curing, the loaded sticks are manually lifted onto a wooden rail framework of four to five levels in the barn, beginning at 2 m (6 feet) above the ground floor and continuing to the top of the barn, sometimes 10 m (30 feet) from the ground (step 10).

After the tobacco leaves are dried, they are taken down from the barn in the reverse process. The dry leaves are stripped from the sticks, loaded into the tobacco press, and compressed into bales; this step is called the baling process (step 11). Each bale weighs around 60–70 kilograms, and these are loaded into trucks for shipment (step 12).

The health hazards that tobacco farmers encounter are similar to other agricultural workers. These include poisoning by pesticides and other chemicals [15,16,17], respiratory effects of tobacco work [18,19], musculoskeletal disorders [20], occupational injuries [21,22], and nicotine poisoning (green tobacco sickness or GTS) [23,24,25].

There have been no studies in Thailand on the MSDs risks faced by Burley tobacco farmers. This study aimed to determine the prevalence and factors associated with MSDs in Thai tobacco farmers during the planting and harvesting.

## 2. Materials and Methods

### 2.1. Subject Recruitment

**Figure 2:**
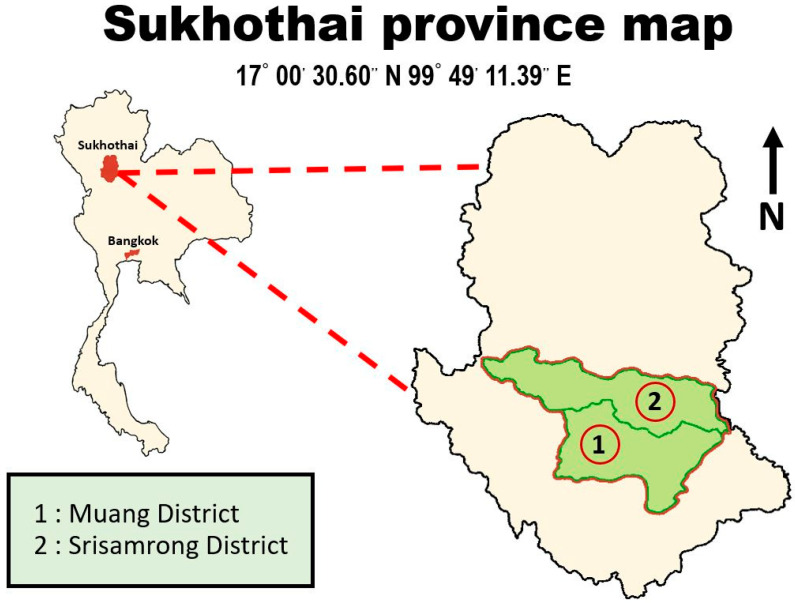
Study area: Sukhothai province (with two districts with Burley tobacco farming).

Two of the nine districts in Sukhothai province, a region in the lower part of northern Thailand, are devoted to Burley tobacco cultivation, as shown in Figure 2. Within the two districts of Srisamrong and Muang, there are 2502 and 1002 registered tobacco family farmers, respectively.

The participants were recruited using systematic random sampling from the available registered tobacco farmers in the area. The inclusion criteria for participants included being 18–80 years of age and had been engaged in at least one task during both the planting and harvesting period during the past year. Potential participants were excluded if they had a preexisting diagnosis of either bone or muscle disease, such as gout, arthritis, rheumatism, osteoporosis, immune deficiency, or menopausal syndrome, or had received surgery for a bone or muscle disorder, including any severe accidents that affected a bone or muscle in the body.

### 2.2. Study Population and Data Collection

A cross-sectional study was conducted with 603 tobacco farmers between December 2016 and May 2017. All subjects gave their informed consent for inclusion before they participated in the study. The study was conducted in accordance with the Declaration of Helsinki, and the protocol was approved by the Ethics Committee of Human Research, Faculty of Public Health, Mahidol University (Protocol No. 173/2559). The farmers were interviewed in person by trained research staff using a questionnaire. The questionnaire consisted of several sections, including demographic information, general health characteristics, musculoskeletal symptoms, and tobacco farming-related activities.

The main focus of the questionnaire addressed musculoskeletal symptoms or discomfort in their body parts using the modified standardized Nordic questionnaire, which has been widely used and validated [26,27,28]. The questionnaire was translated into Thai and then checked by back-translation. Instead of using the standard time frame for symptoms in the past seven days and one year, we asked about the past seven days and three months after both the planting and harvesting periods to ensure that symptoms were related to the specific seasonal variation in tobacco farming-related activities.

After a pilot study to assess face validation, we collected information about tobacco farm work and seasonal tasks to assess seasonal variation in exposures and work habits. Participants were asked to indicate the number of hours per day and the number of days per week they spent performing specific activities during the planting period (November to the end of January) and the harvesting period (February until the end of May). There were six activities during the planting period and six other activities during the harvesting period, about which data were collected. We also collected demographic data, such as age, gender, height, weight, and tobacco farming status. Other questions addressed general health characteristics, including the medical history, smoking and alcohol consumption, exercise, previous surgeries, and past accidents involving bones or muscles.

### 2.3. Data Analyses

The data were validated, coded, and analyzed by SPSS for Windows, Version 18.0 (SPSS (Thailand) Co., Ltd., Bangkok, Thailand). The descriptive results were analyzed using the mean, standard deviation, minimum, and maximum to examine the characteristics of Burley tobacco farmers and the prevalence of MSDs. Univariate logistic regression analysis was used to determine the association between age, gender, BMI, years of work in tobacco farming, and any MSDs [29,30]. Poisson regression models examining work-related risk factors controlled for age, gender, and BMI. The adjusted prevalence ratio (aPR) and corresponding 95% confidence intervals (CIs) were used to report the results. A *p*-value of less than 0.05 was considered statistically significant.

## 3. Results

### 3.1. Characteristics of Subjects

Six hundred and three questionnaires were collected. The demographic data of participants are presented in Table 1. The mean age of the participants was 49.5 (SD 11.61; range 18–79) years. A total of 58.5% of the participants were female, and 74.3% had finished their primary education. The participants had an average body mass index of 24.4 (SD 4.12; range 13.67–40.77) kg/m^2^, which meant 38% of the participants were considered overweight or obese. Regarding the health status of tobacco farmers, 88.2% and 75.1% were non-smokers and non-alcohol drinkers, respectively. Only 9% of the participants exercised more than 3 times per week after working hours.

The tasks performed by tobacco farmers included agricultural activities during the planting and harvesting seasons, as shown in Table 2. Most tobacco farmers performed the seedling (87.9%), plowing (39.1%), digging holes for planting (53.6%), planting (83.3%), and maintenance of the crops (80.3%). The percentage of farmers who plowed with tractors by riding on the vehicles (77.5%) was significantly higher than that of farmers who plowed by hand tractor, which requires walking on the field (22.5%), and the percentage of farmers who dug holes with machines (92.0%) was higher than that of farmers who dug holes manually, but the prevalence of any MSDs was similar between the two groups.

For harvesting, most tobacco farmers performed hand picking leaves at the base, middle, and the top of the tobacco plant in the field, Some farmers (2.8–3.6%) performed hand-picking individually, but most farmers (96.4–97.2%) worked in groups, supporting each other by talking, relaxing, and helping each other. To transport the fresh leaves to the barns, they brought the leaves and put them on wagons. When they arrived at their houses or barns, they had to manually moved the leaves down off the wagons.

During the harvesting season, they usually helped each other with their work for each task: piercing/threading the tobacco leaves, curing the tobacco leaves by hanging them up in the barn and taking them down when they dried, bundling them into sacks, carrying the sacks up to and down from the truck, and transporting the tobacco products to the location where they sold their products.

### 3.2. MSDs in the Planting and Harvesting Season

**Figure 3:**
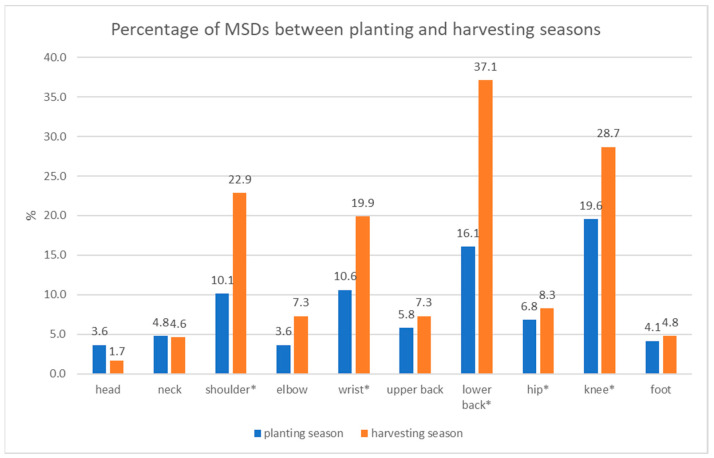
The percentage of MSDs between the planting and harvesting seasons. * = statistically significant difference (*p* < 0.05).

In Figure 3, with regard to MSDs during planting, tobacco farmers reported MSDs in the knee (19.6%), lower back (16.1%), wrists (10.6%), shoulders (10.1%), and hips (6.8%).

In the comparison of MSDs in tobacco farmers between the planting and harvesting seasons, the results showed significantly higher MSDs in the harvesting season, which occurred in the shoulders (*p* = 0.024), wrists (*p* = 0.038), lower back (*p* < 0.001), hips (*p* = 0.007), and knees (*p* < 0.001).

Table 3 univariate analysis showed age, gender, and years of work experience in tobacco farming were factors that were associated with any MSDs during both seasons.

Robust Poisson regression models controlled for age, gender, and BMI were used to determine factors related to the MSDs in the planting and harvesting seasons in Table 4. In the planting season starting with planting seedlings, farmers who worked 4–6 h had significantly lower aPR for any MSD or for pain in the lower back than those working 7 h or more. For plowing, farmers using hand tractors had a significantly higher aPR for knee pain compared to those using tractors they rode on. For the task of digging holes, farmers who performed this task by manual labor had significantly higher aPR for shoulder pain than those using a machine to dig holes. For crop maintenance, spraying pesticides with a backpack sprayer had significantly lower aPR for any MSD and for the knees than spraying pesticides with a vehicle. For cutting the top of the tobacco plant, farmers that cut only 1–3 h had significantly lower aPRs for any MSDs, as well as for MSDs of the wrist, lower back, and knee compared to those doing the task for 7 h or more. 

There was no significant aPR of MSDs in any body parts for hand picking at the top, middle, and base between individual farmer and group work in the harvesting period (not shown in Table 3). The results showed that working in a group could reduce MSDs in different areas of the body; group workers who carried the leaves up to the wagon had a significantly lower aPR of any MSDs and shoulder MSDs, and those who carried the leaves down from the wagon had a significantly lower aPR of any MSDs, shoulder MSDs, and knee MSDs than those of individual farmers.

Regarding piercing/threading the tobacco leaves, group workers had significantly reduced aPRs of any MSDs, wrist MSDs, and lower back MSDs than individual farmers. The pierced leaves were hung up in the barn; group farmers who hung the pierced leaves had significantly lower aPR of MSDs in the shoulder than individual farmers. After curing the leaves, they were brought down from the barn. The group farmers who brought down the cured leaves had significantly lower aPRs of any MSDs and shoulder, wrist, and knee MSDs than individual farmers.

The next step was to make a small bundle of the cured leaves into a bigger size and a sack of leaves. The results showed that group farmers had significantly lower aPRs of any MSDs and shoulder, wrist, lower back, and knee MSDs than individual farmers. The last step was to lift the sacks and bring them down to the vehicle; the group farmers who lifted the sacks had significantly lower aPRs of MSDs in any body parts and shoulder, wrist, and knee MSDs than individual farmers. The group farmers who lifted the sacks down from the vehicles had significantly lower aPRs of MSDs in any location and shoulder, wrist, lower back, and knee MSDs than individual farmers.

## 4. Discussion

Agricultural worker’s health problems include hearing loss, cancer, musculoskeletal disorders (MSDs), pesticide-caused illness, and respiratory illnesses [31], but MSDs seem to be the most widespread problems in agricultural workers because of repetitive lifting and moving of heavy loads, intensive handwork, and working in awkward postures [32,33,34]. Among Thai tobacco farmers, 76.1% were 30–60 years of age, 41.5% were male, and 74.3% completed primary school. Among tobacco farmers in Brazil, 71.7% were 30 to ≥50 years of age, 59.3% were male, and 92.8% had eight years of schooling [20]. The rate of smoking in Thai tobacco farmworkers was 11.8%, which was lower than the rate of smoking (19.8%) in Brazilian tobacco farmworkers. In Thailand, the average number of years of work in tobacco farming was 31 years, ranging from 1 to 62 years, and 82.9% worked for 8 h or less per day. They started working when they were young and continued working until they retired or could not work anymore. In Brazil, 63.7% of them worked less than 20 years. They worked very hard; 87% worked from 9 to 18 h per day. The average farm size of tobacco farming in Thailand was 1.84 acres, compared with 41.5 acres in South Brazil and 240.1 acres in the USA [35].

Regarding MSDs during planting and harvesting, tobacco farmers had significantly higher MSDs in the shoulders, wrists, lower back, hips, and knees when harvesting tobacco leaves than when planting tobacco plants. For harvesting fruit and tree nut crops, the burden of bearing heavy loads, repetitive cutting, and excessive reaching caused pain in the whole body, mainly in the hands, wrists, shoulders, and lower back [34]. The harvesting of tomatoes and lettuce with prolonged stooping, lifting loads of weight, and intensive and repetitive cutting also contributed to discomfort in the hands, wrist, and lower back [34].

Age was significantly associated with any MSDs in tobacco farmers; farmers aged >61 years had significantly higher rates of MSDs than those aged 18–30 years. Female farmers had significantly more MSDs, including those of the lower back, than male farmers. These results were like a study of tobacco farmers in Brazil, which found that farmers aged 30–50 years had significantly more lower back pain than those aged 18–29 years [20]. The lower back pain of male and female tobacco farmers in Brazil was not significantly different, probably because men and women were exposed to different tasks and workloads, but the Thai tobacco farmers were similar depending on their preferences [20]. These farmers worked on several tasks involving the planting, harvesting, curing, packaging, and transportation of the products to the manufacturing companies. The number of years working in tobacco farms significantly increased the aPR of any MSDs, which was similar to Brazil tobacco farmers where farmers who worked ≥ 20 years had significantly higher lower back pain than those working ≤19 years [20]. Both non-smokers and smokers were potentially exposed to nicotine dermally and smokers also inhaled nicotine from form their cigarettes [36,37]. However, we did not investigate the prevalence of green tobacco syndrome symptoms.

During the planting season, when we considered the hours of work within 8 h of regular working hours controlled with age, gender, and BMI, we found that working 4–6 h in seedling had significantly lower aPR of any MSDs and lower back pain than those working 7 h or more. Farmers who performed the task of cutting the top of tobacco plants for 1–3 h had a significantly lower aPR of any MSDs and wrist, lower back, and knee MSDs than those working 7 h or more. The working hours per week of Korean workers resulted in an increased the prevalence of upper (arms, elbow, wrists, and hands) and lower limb (hips, legs, knees, and feet) pain in workers compared with the reference group of weekly working hours when controlling for general and occupational characteristics [38].

For plowing with riding-type tractors and hand tractors, farmers who performed plowing with hand tractors had a significantly higher aPR of knee pain than those who performed the work with riding-type tractors. Prolonged sitting on tractors together with whole-body vibration could lead to discomfort for the operator and an increased risk for lower back, shoulder, neck, knee, and spinal pain [39,40].

During the harvesting season, we compared the individual farmers working alone and farmers hiring other farmers to help with the harvesting tasks because of the short duration of the harvesting period. While working, they talked and supported each other to complete the work more quickly. The results showed that farmers performing hand picking at the top, middle, and base levels did not have a significant difference in the aPR between individual work and group work. This may be because the hand picking of tobacco leaves has to be completed individually, even when working in groups.

The next steps were lifting the tobacco leaves onto the wagon and then unloading the wagon so leaves could be sewn to sticks at the barn, followed by hanging the sewn leaves up in the barn for curing, then bringing the cured leaves down from the barn to be packaged. We found that group work had an aPR of any MSDs and shoulder pain significantly lower than those with individual work for loading and unloading the wagon. It was the same for threading/piercing the leaves and the subsequent steps in curing the tobacco leaves. 

The process of baling the cured tobacco leaves—making small bundles, making bigger bundles, and creating sacks of tobacco leaves—with group work had aPRs of any MSDs and wrist pain that were significantly lower than those for individual work. The same trend of aPRs of any MSDs and shoulder, wrist, and knee pain being significantly lower for those in group work compared to those for individual work was observed for the transportation of finished packs of tobacco leaves to the manufacturing company.

Work-related musculoskeletal disorders (MSDs) have a multifactorial etiology, including physical stressors and psychosocial risk factors, such as job strain, social support at work, and job dissatisfaction [41]. Psychosocial factors have been shown to impact the increase and exacerbation of MSDs [42]. When farmers have much work to do above their ability, they tend to have stress. The stress could increase blood pressure and increase pressure in the joints, specifically on tendons, ligaments, and nerves. The stress could also increase muscle tension, which may cause excessive use of force during certain activities and movements. If farmers work in a group, they can talk to, cheer up, and support each other, and they can work with less stress and less pain in their body parts [43]. The results from a Korean study found that workers under high job stress and low social support had significantly higher upper limb (arms, elbow, wrists, and hands) and lower limb (hips, legs, knees, and feet) pain, in both males and females, compared with those with low job stress and high social support [38].

The limitations of our study include its cross-sectional nature and potential recall bias [44]. In this study, no data were collected on psychosocial factors, such as social support, job demands, or job satisfaction.

## 5. Conclusions

Tobacco work involves manual labor during both the planting and harvesting seasons. Farmers had significantly higher MSDs in the shoulders, wrists, lower back, hips, and knees in the harvesting season than in the planting season. The results showed that the high prevalence of MSDs in tobacco workers was the same as in other agricultural work. Reduced hours of work reduced the risk of MSDs during the planting season. Activities that used machines rather than manual labor reduced the MSDs in tobacco farmers. Working in a group is better than individual work during the harvesting season since it reduced the prevalence of MSDs. To further reduce MSDs, appropriate equipment needs to be provided to help farmers reduce muscle stress. In the future, new ergonomic tools for tobacco farming need to be developed and provided for each gender of tobacco farmers. 

## Figures and Tables

**Table 1:** Demographic characteristics of Thai Burley tobacco farmers (*n* = 603).

Variables		Thai Burley Tobacco Farmers *n* (%)
**Age** (mean ± SD): 49.5 ± 11.6 years		
	18–30	42 (7.0)
	31–60	459 (76.1)
	≥61	102 (16.9)
**Gender**		
	Male	250 (41.5)
	Female	353 (58.5)
**Body Mass Index (BMI)** (mean ± SD): 24.4 ± 4.1 kg/m^2^		
	<18.50 (Underweight)	19 (3.1)
	18.5–24.9 (Normal)	355 (58.9)
	≥25.00 (Overweight or Obese)	229 (38.0)
**Educational level**		
	Primary school	448 (74.3)
	Secondary school or higher	155 (25.7)
**Marital status**		
	Single	78 (12.9)
	Married	492 (81.6)
	Widowed/divorced	33 (5.5)
**Income** (Thai Baht)		
	<100,000	410 (68.0)
	≥100,000	193 (32.0)
**Alcohol consumption behavior**		
	Current drinking	150 (24.9)
	Non-drinking	453 (75.1)
**Smoking behavior**		
	Current smoking	71 (11.8)
	Non-smoking	532 (88.2)
**Exercise**		
	Yes (more than 3 times/week)	54 (9.0)
	No	549 (91.0)
**Year of work in tobacco farming (year)**		
	Min–Max	1–62
	Mean (SD)	30.98 (12.65)
**Working time in planting season (h/day)**		
	1–3 h	8 (1.3)
	4–6 h	197 (32.7)
	7 or more than	398 (66.0)
**Working time in harvesting season (h/day)**		
	1–3 h	21 (3.5)
	4–6 h	161 (26.7)
	7 or more	421 (69.8)

**Table 2:** Task performed by tobacco farmers during the planting and harvesting season (*n* = 603).

Task		Thai Burley Tobacco Farmers *n* (%)	Prevalence of MSDs *n* (%)
**Planting Season**			
Seedling (*n* = 530)			
	1–3 h	95 (17.9)	47 (49.5)
	4–6 h	267 (50.4)	117 (43.8)
	7 or more	168 (31.7)	96 (57.1)
Plowing (*n* = 236)			
	Tractor (riding on a vehicle)	183 (77.5)	70 (38.3)
	Hand tractor (controlled by walking)	53 (22.5)	21 (39.6)
Digging holes for planting (*n* = 323)			
	Machine digging	297 (92.0)	135 (45.5)
	Manual digging	26 (8.0)	13 (50.0)
Planting: filling the holes with water (*n* = 502)			
	1–3 h	116 (23.1)	54 (46.6)
	4–6 h	245 (48.8)	126 (51.4)
	7 or more	141 (28.1)	70 (49.6)
Crop maintenance: cutting the top of the tobacco plant or topping (*n* = 484)			
	1–3 h	74 (15.3)	31 (41.9)
	4–6 h	254 (52.5)	113 (44.5)
	7 or more	156 (32.2)	98 (62.8)
**Harvesting Season**			
Hand-picking			
	Base or Lower (*n* = 576)		
	- Individual	16 (2.8)	11 (68.8)
	- Group work	560 (97.2)	352 (62.9)
	Middle (*n* = 577)		
	- Individual	21 (3.6)	15 (71.4)
	- Group work	556 (96.4)	349 (62.8)
	Top (*n* = 576)		
	- Individual	21 (3.6)	15 (71.4)
	- Group work	555 (96.4)	349 (62.9)
Carrying (hauling from the field to the barn)			
	Putting it on the wagon (*n* = 417)		
	- Individual	78 (18.7)	54 (69.2)
	- Group work	339 (81.3)	183 (54.0)
	Carrying it down to the barn (*n* = 432)		
	- Individual	67 (15.5)	52 (77.6)
	- Group work	365 (84.5)	193 (52.9)
Threading/piercing the tobacco leaves (*n* = 529)			
	- Individual	24 (4.5)	21 (87.5)
	- Group work	505 (95.5)	320 (63.4)
Curing by hanging			
	Hanging up (*n* = 363)		
	- Individual	120 (33.1)	75 (62.5)
	- Group work	243 (66.9)	115 (47.3)
	Climbing down with product (*n* = 407)		
	- Individual	96 (23.6)	68 (70.8)
	- Group work	311 (76.4)	152 (48.9)
Baling			
	Bundle (small size) (*n* = 520)		
	- Individual	72 (13.8)	55 (76.4)
	- Group work	448 (86.2)	275 (61.4)
	Bundle (medium size) (*n* = 454)		
	- Individual	97 (21.4)	70 (72.2)
	- Group work	357 (78.6)	202 (56.6)
	Sack (large size) (*n* = 427)		
	- Individual	82 (19.2)	70 (85.4)
	- Group work	345 (80.8)	165 (47.8)
Transportation			
	Lifting it onto the vehicle (*n* = 453)		
	- Individual	38 (8.4)	32 (84.2)
	- Group work	415 (91.6)	232 (55.9)
	Lifting it down from the vehicle (*n* = 443)		
	- Individual	37 (8.4)	30 (81.1)
	- Group work	406 (91.6)	226 (55.7)

**Table 3:** Univariate analysis of factors associated with any MSDs.

Task			Any MSDs				
		Planting Season			Harvesting Season		
		PR	95% CI	*p*-Value	PR	95% CI	*p*-Value
**Age (Year)** **(Ref = age 18–30 years)**							
	31–60	2.36	1.31, 4.24	0.004 *	1.38	0.98, 1.94	0.062
	≥61	2.75	1.50, 5.01	0.001 *	1.76	1.24, 2.48	0.001 *
**Gender** **(Ref = male)**							
	Female	1.25	1.05, 1.48	0.011 *	1.16	1.02, 1.31	0.024 *
**BMI (kg/m^2^)** **(Ref = 18.5–24** **.** **9 (normal))**							
	<18.50 (Underweight)	1.08	0.70, 1.68	0.732	1.21	0.91, 1.60	0.193
	≥25.00 (Overweight and Obese)	1.06	0.90, 1.25	0.507	1.11	0.99, 1.26	0.080
**Years of work in tobacco farming (year)**		1.02	1.01, 1.02	<0.001 *	1.01	1.01, 1.02	<0.001 *

Note: * statistically significant (*p* < 0.05), abbreviations: PR, prevalence ratio; 95% CI, 95% confidence interval.

**Table 4:** Factors related to MSDs in the planting and harvesting seasons using Robust Poisson Regression adjusted by age, gender, and BMI.

Task			Any MSDs			Shoulder			Wrist			Lower Back			Knee		
			aPR	95% CI	*p*-Value	aPR	95% CI	*p*-Value	aPR	95% CI	*p*-Value	aPR	95% CI	*p*-Value	aPR	95% CI	*p*-Value
**Planting Season**																	
**Seedling** **(Ref ≥ 7 h)**		4–6 h	0.76	0.63, 0.91	0.003 *	0.99	0.52, 1.88	0.973	0.77	0.45, 1.32	0.342	0.63	0.41, 0.97	0.035 *	0.75	0.51, 1.11	0.753
		1–3 h	0.84	0.66, 1.07	0.156	1.93	0.96, 3.89	0.066	0.90	0.45, 1.81	0.898	0.62	0.34, 1.13	0.120	1.04	0.67, 1.63	0.857
**Plowing** **(Ref = Riding-type tractor)**		Using hand tractor	1.08	0.74, 1.58	0.682	1.80	0.79, 4.08	0.161	1.19	0.52, 2.72	0.673	1.13	0.54, 2.35	0.746	2.29	1.19, 4.39	0.013 *
**Digging holes (Ref = machine)**		Manual	1.08	0.73, 1.60	0.692	2.92	1.49, 5.72	0.002 *	0.55	0.15, 2.00	0.363	0.71	0.24, 2.14	0.544	1.31	0.62, 2.78	0.474
**Planting** **(Ref ≥ 7 h)**		4–6 h	1.00	0.82, 1.23	0.973	0.87	0.46, 1.62	0.649	0.60	0.34, 1.05	0.075	0.96	0.58, 1.58	0.870	0.93	0.62, 1.42	0.744
		1–3 h	0.89	0.69, 1.15	0.372	1.26	0.65, 2.42	0.494	0.53	0.26, 1.10	0.089	1.08	0.61, 1.91	0.783	0.86	0.52, 1.42	0.555
**Crop maintenance (Ref = Spraying by vehicle)**		Spraying with backpack	0.77	0.64, 0.92	0.005 *	0.99	0.58, 1.69	0.965	0.82	0.47, 1.42	0.479	0.50	0.32, 0.78	0.002 *	0.61	0.41, 0.90	0.012 *
**Topping the tobacco plant** **(Ref ≥ 7 h)**		4–6 h	0.74	0.62, 0.89	0.001 *	1.06	0.58, 1.94	0.852	1.21	0.69, 2.12	0.501	1.08	0.71, 1.66	0.718	0.79	0.54, 1.17	0.244
		1–3 h	0.68	0.50, 0.91	0.010 *	1.46	0.69, 3.08	0.324	0.13	0.02, 0.97	0.047 *	0.40	0.16, 0.98	0.045 *	0.51	0.26, 0.99	0.048 *
**Harvesting Season**																	
**Carrying** from the field **(Ref = individual work)**																	
	Loading	Group work	0.81	0.66, 0.98	0.029 *	0.60	0.40, 089	0.012 *	0.74	0.94, 1.39	0.351	0.88	0.63, 1.22	0.434	0.79	0.53, 1.20	0.273
	Unloading		0.69	0.58, 0.83	<0.001 *	0.51	0.35, 0.75	<0.001 *	0.67	0.37, 1.22	0.188	0.81	0.58, 1.12	0.201	0.60	0.40, 0.88	0.009 *
**Piercing/Threading (Ref = individual work)**		Group work	0.72	0.61, 0.85	<0.001 *	0.63	0.35, 1.14	0.124	0.32	0.24, 0.44	<0.001 *	0.61	0.43, 0.88	0.008 *	0.71	0.43, 1.16	0.172
**Curing in the barn (Ref = individual work)**																	
	Hanging up	Group work	0.83	0.68, 1.01	0.070	0.56	0.37, 0.83	0.004 *	1.21	0.64, 2.30	0.553	1.06	0.77, 1.46	0.705	0.68	0.46, 1.01	0.053
	Climbing down		0.73	0.61, 0.88	0.001 *	0.65	0.44, 0.97	0.033 *	0.55	0.34, 0.89	0.015 *	0.76	0.56, 1.03	0.080	0.57	0.39, 0.84	0.004
**Baling (Ref = individual work)**																	
	Small size	Group work	0.83	0.71, 0.96	0.014 *	0.70	0.47, 1.04	0.074	0.50	0.35, 0.71	<0.001 *	0.85	0.63, 1.16	0.311	0.76	0.55, 1.05	0.099
	Medium size		0.79	0.68, 0.93	0.003 *	0.51	0.36, 0.73	<0.001 *	0.43	0.30, 0.63	<0.001 *	0.75	0.58, 0.98	0.033 *	0.83	0.58, 1.18	0.295
	Large size		0.55	0.47, 0.65	<0.001 *	0.36	0.25, 0.51	<0.001 *	0.46	0.27, 0.78	0.004	0.53	0.40, 0.71	<0.001 *	0.31	0.21, 0.44	<0.001 *
**Transportation (Ref = individual work)**																	
	Loading	Group work	0.67	0.56, 0.79	<0.001 *	0.45	0.30, 0.68	<0.001 *	0.39	0.23, 0.67	0.001 *	0.72	0.50, 1.04	0.082	0.54	0.36, 0.83	0.005 *
	**Unloading**		**0.70**	**0.58, 0.85**	<0.001 *	**0.49**	**0.32, 0.75**	0.001 *	**0.37**	**0.22, 0.65**	<0.001 *	**0.72**	**0.50, 1.03**	0.074	**0.61**	**0.38, 0.97**	0.036 *

Note: * statistically significant (*p* < 0.05), abbreviations: aPR, adjusted prevalence ratio; 95% CI, 95% confidence interval.

## Data Availability

Not applicable.

## References

[1] International Labour Organization (ILO) Agriculture: A Hazardous Work. https://www.ilo.org/safework/areasofwork/hazardous-work/WCMS_110188/lang--en/index.htm.

[2] Davis K.G., Kotowski S.E. (2007). Understanding the ergonomic risk for musculoskeletal disorders in the United States agricultural sector. Am. J. Ind. Med..

[3] Bernard B.P. (1997). Musculoskeletal Disorders and Workplace Factors: A Critical Review of Epidemiological Evidence for Work-Related Musculoskeletal Disorders of the Neck, Upper Extremity and Low Back.

[4] Walker-Bone K., Palmer K.E. (2003). Musculoskeletal disorders in farmers and farm workers. Occup. Med..

[5] Tamene A., Mulugeta H., Ashenafi T., Thygerson S.M. (2020). Musculoskeletal disorders and associated factors among vehicle repair workers in Hawassa City, Southern Ethiopia. J. Environ. Public Health.

[6] Spallek M., Kuhn W., Uibel S., Mark A.V., Quarcoo D. (2010). Work-related musculoskeletal disorders in the automotive industry due to repetitive work—Implications for rehabilitation. J. Occup. Med. Toxicol..

[7] National Statistics Office Summary of Informal Employment Worker Survey. http://www.nso.go.th/sites/2014/DocLib13/%e0%b8%94%e0%b9%89%e0%b8%b2%e0%b8%99%e0%b8%aa%e0%b8%b1%e0%b8%87%e0%b8%84%e0%b8%a1/%e0%b8%aa%e0%b8%b2%e0%b8%82%e0%b8%b2%e0%b9%81%e0%b8%a3%e0%b8%87%e0%b8%87%e0%b8%b2%e0%b8%99/Informal_work_force/2564/summary_64.pdf.

[8] Chanprasit C., Kaewthummanukul T. (2010). Occupational health hazards, work-related illness and injury, work behaviors among informal workforce: Case study in baby corn planting farmer group. Public Health J. Burapha Univ..

[9] Sombatsawat E., Luangwilai T., Ong-artborirak P., Siriwong W. (2019). Musculoskeletal disorders among rice farmers in Phimai District, Nakhon Ratchasima Province, Thailand. J. Health Res..

[10] Prommawa N., Laohasiriwong W., Nilvarangkul K. (2019). Musculoskeletal disorders and quality of life of sugarcane farmers in the northeast of Thailand: A cross-sectional analytical study. J. Clin. Diagn. Res..

[11] Meksawi S., Tangtrakulwanich B., Chongsuvivatwong V. (2012). Musculoskeletal problems and ergonomic risk assessment in rubber tappers: A community-based study in southern Thailand. Int. J. Ind. Ergon..

[12] Yogyorn D., Slatin C., Siriruttaanapruk S., Woskie S., Chantian T., Chaladlerd T., Chaladlerd C., Kongtip P. (2021). Estimating of the costs of nonfatal occupational injuries and illnesses in agricultural works in Thailand. J. Public Health Policy.

[13] Tobacco Control Research and Knowledge Management Center (TRC) Thailand Tobacco Control Country Profile. https://untobaccocontrol.org/impldb/wp-content/uploads/reports/annextwothai.pdf.

[14] Thailand Tobacco Monopoly (TTM) (2014). Thailand Tobacco Monopoly: Annual Report. http://www.thaitobacco.or.th/th/wp-content/uploads/2015/08/ebook-annual-report2014.pdf.

[15] Lonsway J.A., Byers M.E., Dowla H.A., Panemangalore M., Antonious G.F. (1997). Dermal and respiratory exposure of mixers/sprayers to acephate, methamidophos, and endosulfan during tobacco production. Bull. Environ. Contam. Toxicol..

[16] Curwin B.D., Hein M.J., Sanderson W.T., Nishioka M., Buhler W. (2003). Acephate exposure and decontamination on tobacco harvesters’ hands. J. Expo. Anal. Environ. Epidemiol..

[17] Cornwall J.E., Ford M.L., Liyanage T.S., Win Kyi Daw D. (1995). Risk assessment and health effects of pesticides used in tobacco farming in Malaysia. Health Policy Plan..

[18] Ghosh S.K., Parikh J.R., Gokani V.N., Rao M.N., Kashyap S.K., Chatterjee S.K. (1979). Studies on occupational health problems in agricultural tobacco workers. J. Soc. Occup. Med..

[19] Osim E.E., Musabayane C.T., Mufunda J. (1998). Lung function of Zimbabwean farm workers exposed to flue curing and stacking of tobacco leaves. S. Afr. Med. J..

[20] Meucci R.D., Fassa A.G., Faria N.M.X., Fiori N.S. (2015). Chronic low back pain among tobacco farmers in southern Brazil. Int. J. Occup. Environ. Health.

[21] Pugh K.J., Pienkowski D., Gorczyca J.T. (2000). Musculoskeletal trauma in tobacco farming. Orthopedics.

[22] Struttmann T.W., Reed D.K. (2002). Injuries to tobacco farmers in Kentucky. South. Med. J..

[23] Arcury T.A., Quandt S.A., Preisser J.S., Bernert J.T., Norton D., Wang J. (2003). High levels of transdermal nicotine exposure produce green tobacco sickness in Latino farmworkers. Nicotine Tob. Res..

[24] Curwin B.D., Hein M.J., Sanderson W.T., Nishioka M.G., Buhler W. (2005). Nicotine exposure and decontamination on tobacco harvesters’ hands. Ann. Occup. Hyg..

[25] Saleeon T., Siriwong W., Maldonado-Perez H.L., Robson M.G. (2015). Green tobacco sickness among Thai traditional tobacco farmers, Thailand. Int. J. Occup. Environ. Med..

[26] Kuorinka I., Jonsson B., Kilbom A., Vinterberg H., Biering-Sorensen F., Andersson G., Jorgensen K. (1987). Standardised Nordic questionnaires for the analysis of musculoskeletal symptoms. Appl. Ergon..

[27] Udom C., Janwantanakul P., Kanlayanaphotporn R. (2016). The prevalence of low back pain and its associated factors in Thai rubber farmers. J. Occup. Health.

[28] Deros B.M., Ali M.H., Mohamad D., Daruis D.D.I. (2016). Ergonomic risk assessment on oil palm industry workers. Iran. J. Public Health.

[29] Jane R., Meena M.L., Dangayach G.S., Bhardwaj A.K. (2018). Risk factors for musculoskeletal disorders in manual harvesting farmers of Rajasthan. Ind. Health.

[30] Patil S.A., Kadam Y.R., Mane A.S., Gore A.D., Dhumale G.B. (2018). The Prevalence and Health Impact of Musculoskeletal Disorders among Farmers. Med. J. DY Patil Vidyapeeth.

[31] Kirkhorn S.R., Schenker M.B. (2002). Current health effects of agricultural work: Respiratory disease, cancer, reproductive effects, musculoskeletal injuries, and pesticide—Related illnesses. J. Agric. Saf. Health.

[32] Meyers J.M., Miles J.A., Faucett J., Janowitz I., Tejeda D.G., Kabashima J.N. (1997). Ergonomics in agriculture: Workplace priority setting in the nursery industry. Am. Ind. Hyg. Assoc. J..

[33] Fathallah F.A., Miller B.J., Miles J.A. (2008). Low Back Disorders in Agriculture and the Role of Stooped Work: Scope, Potential Interventions, and Research Needs. J. Agric. Saf. Health.

[34] Fathallah F.A. (2010). Musculoskeletal disorders in labor-intensive agriculture. Appl. Ergon..

[35] Riquinho D.L., Hennington E.A. (2012). Health, environment and working conditions in tobacco cultivation: A review of the literature. Ciência Saúde Coletiva.

[36] Kongtip P., Trikunakornwong A., Chantanakul S., Loosereewanich P., Rojanavipart P. (2009). Assessment of nicotine dermal contact and urinary cotinine of tobacco processing workers. J. Med. Assoc. Thai..

[37] Trikunakornwong A., Kongtip P., Chantanakul S., Loosereewanich P., Rojanavipart P. (2009). Assessment of nicotine inhalation exposure and urinary cotinine of tobacco processing workers. J. Med. Assoc. Thai..

[38] Lee J.-G., Kim G.H., Jung S.W., Kim S.W., Lee J.-H., Lee K.-J. (2018). The association between long working hours and work-related musculoskeletal symptoms of Korean wage workers: Data from the fourth Korean working conditions survey (a cross-sectional study). Ann. Occup. Environ. Med..

[39] Servadio P., Marsili A., Belfiore N.P. (2007). Analysis of driving seat vibrations in high forward speed tractors. Biosyst. Eng..

[40] Benos L., Tsaopoulos D., Bochtis D. (2020). A Review on Ergonomics in Agriculture. Part II: Mechanized Operations. Appl. Sci..

[41] Menzel N.N. (2007). Psychosocial factors in musculoskeletal disorders. Crit. Care Nurs. Clin. N. Am..

[42] Jaffar N.A., Rahman M.N.A. (2017). Review on risk factors related to lower back disorders at workplace. IOP Conf. Ser. Mater. Sci. Eng..

[43] Canadian Center for Occupational Health and Safety Musculoskeletal Disorders—Psychosocial Factors. https://www.ccohs.ca/oshanswers/psychosocial/musculoskeletal.html.

[44] Jenkins P., Earle-Richardson G., Slingerland D.T. (2002). Time dependent memory decay. Am. J. Ind. Med..

